# From waste to wellness: Citrus by-products as nutritional and immunological enhancers in aquaculture

**DOI:** 10.1016/j.fochx.2026.103523

**Published:** 2026-01-12

**Authors:** Yu Wang, Meng-Ze Nie, Ping Shi, Yuan-Sen Liu, Wei-Bing Lan, Zi-Ru Dai, Muhammad Adeel, Hafiz Umer Javed

**Affiliations:** a Guangxi Zhuang Autonomous Region Engineering Research Center of Marine Food Nutrition and Processing Technology Innovation, Guangxi College and University Key Laboratory of High-value Utilization of Seafood and Prepared Food in Beibu Gulf, Beibu Gulf University, Qinzhou 535011, China; b Qinzhou Key Laboratory of Food Flavor Analysis and Control, Beibu Gulf University, Qinzhou 535011, China; c Beijing Normal University at Zhuhai, Zhuhai, Guangdong 519087, China

**Keywords:** Citrus by-products, Extraction techniques, Bioactive compounds, Fish health

## Abstract

The aquaculture industry plays a vital role in global protein supply; however, it faces significant challenges, including escalating feed costs, disease outbreaks, and concerns over environmental sustainability. Citrus by-products offer a valuable and underutilized renewable resource for developing functional aquafeeds. Green extraction technologies, particularly ultrasound-assisted extraction, have demonstrated high efficiency in recovering bioactive compounds, increasing phenolic yields by more than 30 % while substantially reducing solvent consumption. The inclusion of citrus-derived bioactive in aquafeeds enhances growth performance, immune function, and disease resistance in aquatic species. For example, dietary supplementation with lemon peel extracts improved survival in Nile tilapia to over 80 %, while bitter orange essential oil increased growth rate and modulated immune-related gene expression in common carp. Despite these promising results, bioavailability, metabolic pathways, and species-specific responses remain insufficiently understood. This review synthesizes advances in green extraction and functional applications of citrus bioactives, identifying critical research gaps to guide sustainable aquaculture innovation.

## Highlights

• Citrus by-products enhance fish growth, immunity, and disease resistance.
• Essential oils from citrus peels improve metabolic regulation in fish.
• Green extraction technologies can efficiently extract bioactive compounds from citrus waste.
• Citrus-derived pectin, oils, and phenolics improve fish health, with focus on mechanisms.
• Citrus by-products support sustainable aquaculture by reducing waste and enhancing fish performance.

## Graphical abstract

**Figure:**
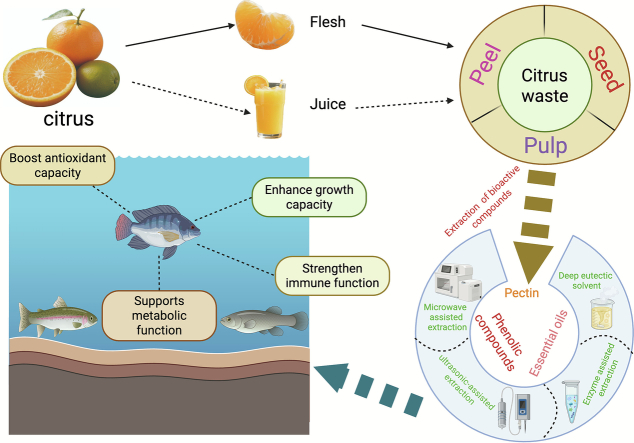

## 1 Introduction

**Fig. 1:**
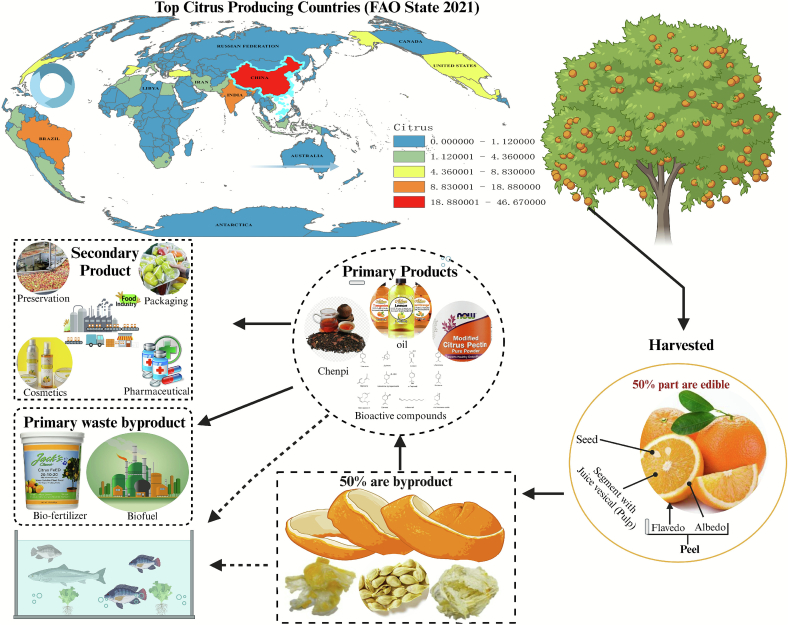
Citrus production and by-products utilization.

Citrus fruits, including oranges, lemons, limes, grapefruits, pomelos, mandarins, bitter oranges, and kumquats, are among the most widely cultivated and consumed fruits worldwide, playing a vital role in global agriculture, nutrition, and health (Richa et al., 2023). They are rich sources of bioactive compounds such as ascorbic acid, flavonoids, limonoids, carotenoids, and polyphenols, which contribute to their antioxidant, antimicrobial, and immunomodulatory properties (Lado et al., 2018; Suri et al., 2022). These bioactive compounds not only provide significant health benefits for humans, including enhanced immune function and reduced risk of chronic diseases such as cardiovascular disorders and cancer, but also have potential applications in various industries, including food, pharmaceuticals, cosmetics, and aquaculture (Fig. 1).

The global citrus industry is dominated by top producing countries such as China, Brazil, India, Mexico and Spain, which together account for a substantial portion of total citrus production (FAO, 2021). However, the citrus processing industry generates vast amounts of waste, with nearly 50 % of the total fruit weight consisting of by-products such as peels, seeds, and pulp (Durmus et al., 2024). These by-products, while often discarded, are rich in bioactive compounds with numerous potential applications. As illustrated in Fig. 1, citrus-producing regions contribute significantly to citrus waste, which, if utilized efficiently, could serve as a valuable resource for multiple industries. Unfortunately, an estimated 10 million tons of citrus waste are generated annually, leading to environmental concerns and economic losses, such waste is commonly discarded in landfills, where its decomposition releases greenhouse gases, including carbon dioxide and methane, thereby intensifying environmental burdens. The high oxygen demand of its organic constituents poses a risk to groundwater quality, while the antimicrobial activity and intrinsic acidity of phenolic compounds can disrupt soil ecosystems. At the same time, the disposal of this biomass represents a substantial loss of recoverable bioresources, leading to avoidable economic costs and undermining opportunities for circular and sustainable valorization (Kim et al., 2022; Suri et al., 2022). The sustainable valorization of these by-products has thus become a focus of research, with efforts directed toward extracting valuable bioactive compounds and developing innovative applications.

Citrus peels, in particular, contain high concentrations of flavonoids, phenolic acids, essential oils (such as *d*-limonene, *β*-caryophyllene, and linalool), pectin, and dietary fibers, all of which possess strong antioxidant, antimicrobial, and hypoglycemic properties (Guo et al., 2024; Yun & Liu, 2022). In recent years, significant progress has been made in extraction technologies aimed at maximizing the yield and functionality of these bioactive compounds. Notably, ultrasound-assisted extraction (UAE) and microwave-assisted extraction (MAE) have shown promise in enhancing extraction efficiency, reducing solvent consumption, and preserving compound integrity (Ciriminna et al., 2024). Additionally, the use of Deep Eutectic Solvents (DES) has emerged as a novel and environmentally friendly approach, offering tunable physicochemical properties and biodegradability. DES-based extraction enables the selective and efficient recovery of polar bioactives without the environmental drawbacks associated with traditional organic solvents. These advancements in green extraction technologies are essential for promoting the sustainable valorization of citrus by-products and expanding their industrial applications.

One of the most promising applications of citrus by-products is in aquaculture as feed additives, where their bioactive-rich composition offers substantial benefits in fish growth, immunity, and disease resistance. As aquaculture expands to meet increasing global demand, it faces challenges such as feed sustainability, disease outbreaks, and rising production costs. Traditional fish feeds rely on fishmeal and synthetic additives, which contribute to overfishing and increase the industry's ecological footprint. Citrus by-products provide a natural, cost-effective alternative by acting as growth promoters, immune boosters, and antimicrobial agents (Kesbiç et al., 2022; Saeed et al., 2024). Their high content of flavonoids, phenolic acids, and essential oils enhances metabolism, gut microbiota composition, and nutrient absorption, leading to improved growth performance. Additionally, essential oils such as *d*-limonene and linalool exhibit strong antimicrobial properties, protecting fish against pathogens like *Edwardsiella tarda* and *Aeromonas hydrophila*, thereby reducing the need for synthetic antibiotics (Baba et al., 2016; Kesbiç et al., 2022). The presence of carotenoids in citrus peels also enhances skin pigmentation in ornamental and farmed fish, improving their commercial value. Moreover, citrus-derived compounds improve serum biochemistry, hematological profiles, and oxidative stress markers, leading to better health resilience and stress tolerance in aquaculture species such as tilapia (*Oreochromis* spp.), carp (*Cyprinus carpio*), trout (*Oncorhynchus mykiss*), and seabass (*Dicentrarchus labrax*) (Kesbiç et al., 2022; Zhang et al., 2023). The integration of citrus by-products into fish diets supports sustainable aquaculture development by reducing dependency on fishmeal, mitigating environmental waste, and promoting eco-friendly feed formulations.

Although substantial progress has been made in characterizing the types, functions, and applications of bioactive compounds in citrus by-products and green extraction technologies continue to advance, current research remains largely focused on individual extraction techniques or single bioactive compounds. While each green extraction methods offer distinct advantages and limitations, systematic and comparative analyses of their extraction efficiencies are still scarce. Moreover, evaluations of citrus-derived bioactives as aquafeed additives are often conducted under specific experimental conditions, with limited consideration of critical variables such as citrus variety, supplementation dosage, and fish species-specific response. To overcome these limitations, this review adopts an integrative perspective that simultaneously examines extraction performance, bioactive composition, and biological functionality. Accordingly, this review provides a comprehensive overview of recent advances in the utilization of citrus by-products as functional feed additives in aquaculture, with particular emphasis on key bioactive constituents, including flavonoids, phenolic acids, pectin, and essential oils, and their roles in modulating fish growth, immunity, and overall health, with explicit consideration of dose-dependent and species-specific effects. Furthermore, recent developments in innovative and environmentally friendly extraction technologies, such as UAE, MAE, and DES, are critically compared in terms of efficiency and sustainability, offering a unified framework for selecting optimal extraction strategies. Finally, the integration of citrus by-products into aquafeeds is discussed within a circular economy framework, highlighting their potential to simultaneously reduce food waste, decrease reliance on synthetic additives, and enhance the environmental sustainability of aquaculture systems.

## 2 Background and valorization of citrus by-products in aquaculture systems

The surplus of citrus waste, often discarded on land due to limited disposal options, was historically a neglected resource. As this organic matter decomposed, it contributed to nutrient enrichment in the soil. Over time, citrus by-products were identified as a potential alternative to traditional grains in animal feed (Panwar et al., 2021), and research into their valorization for aquaculture has grown steadily.

**Fig. 2:**
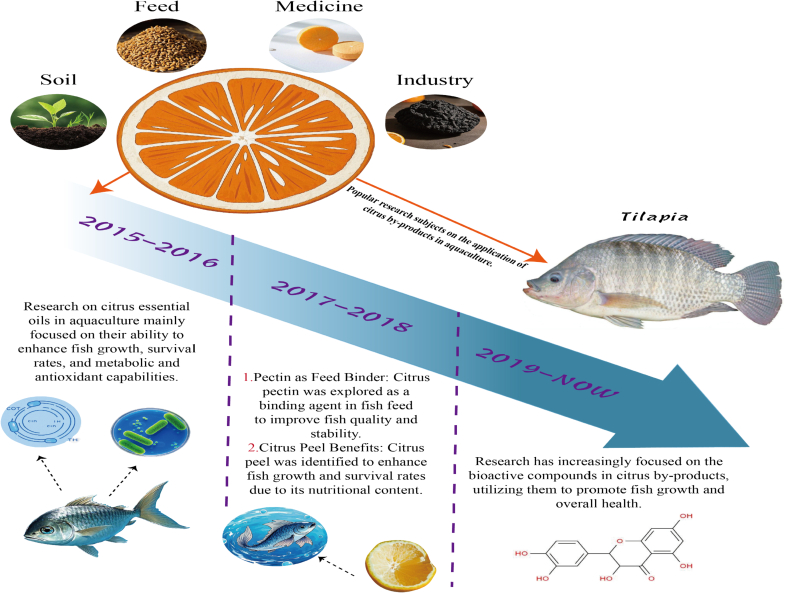
Development of citrus by-products in the aquaculture.

Fig. 2 illustrated the applications and developmental background of citrus by-products in aquaculture. Since 2015, the potential of citrus waste in aquaculture has attracted increasing scientific attention, with numerous studies confirming its function effectiveness. Early investigation conducted between 2015 and 2016 primarily focused on citrus essential oils, highlighting their considerable value as aquafeed additives. These studies indicated that essential oils derived from sweet orange and lemon, when incorporated into tilapia diet, significantly enhanced immune parameters such as lysozyme and peroxidase activity, improved hematological and biochemical markers including hemoglobin, blood glucose, and triglyceride levels, and increased resistance to bacterial infections such as *Streptococcus iniae* and *Edwardsiella tarda* (Acar et al., 2015; Baba et al., 2016). Collectively, these effects translated into improved survival rates in tilapia, supporting the potential of citrus essential oils as natural and sustainable alternatives to conventional antibiotics in aquaculture.

After 2017, research attention expanded to other citrus by-products beyond essential oils. Citrus pectin was found not only to promote embryonic evolution in zebrafish but also to enhance the growth performance of common carp, improving skin mucus immune parameters and serum immunoglobulin levels, highlighting its potential as a high-quality feed additive (Dananjaya et al., 2020; Hosseini et al., 2020). Concurrently, citrus peel itself was applied as a feed additive. Feeding gilthead seabream (*Sparus aurata*) with it resulted in significant weight gain, improved humoral immunity, enhanced phagocytic capacity of head-kidney leukocytes, and upregulation of immune-related genes, all without inducing liver damage at appropriate inclusion levels (Basto-Silva et al., 2022). These findings collectively highlight the expanding functional applications of diverse citrus by-products in aquafeed formulations.

More recently, research on citrus waste in aquaculture has expanded to explore a broader range of bioactive compounds. Citrus peel extracts enriched with phenolic and ketonic compounds have been reported to increase red blood cell count, white blood cell count, hemoglobin levels, and intestinal villus development in Caspian white fish (*Rutilus frisii kutum*), while also reducing juvenile mortality in tilapia caused by *Aeromonas hydrophila* infection (Samavat et al., 2019; Silva et al., 2024). Furthermore, they enhance the activity of liver enzymes such as malate dehydrogenase, citrate synthase, and superoxide dismutase in Rohu (*Labeo rohita*) fingerlings reared under low-temperature conditions, thereby improving the fish's metabolic and antioxidant capacities (Tejaswini et al., 2024). These recent findings highlight the growing potential of citrus-derived bioactives to support fish health, immunity, and overall aquaculture productivity.

This evolving research trajectory confirms citrus by-products as a multi-functional source of aquafeed additives. However, realizing their full potential in practical applications critically depends on the efficient recovery of the bioactive compounds responsible for these benefits. The efficacy, safety, and economic viability of the final product are fundamentally determined by the extraction process employed. Conventional methods often face limitations in selectivity, yield, or environmental impact, underscoring the necessity for green extraction techniques. These advanced methods are designed to maximize extraction efficiency while minimizing energy use, solvent consumption, and ecological impact, thereby aligning the valorization of citrus waste with the principles of sustainable and circular aquaculture.

## 3 Innovative green extraction technology

**Fig. 3:**
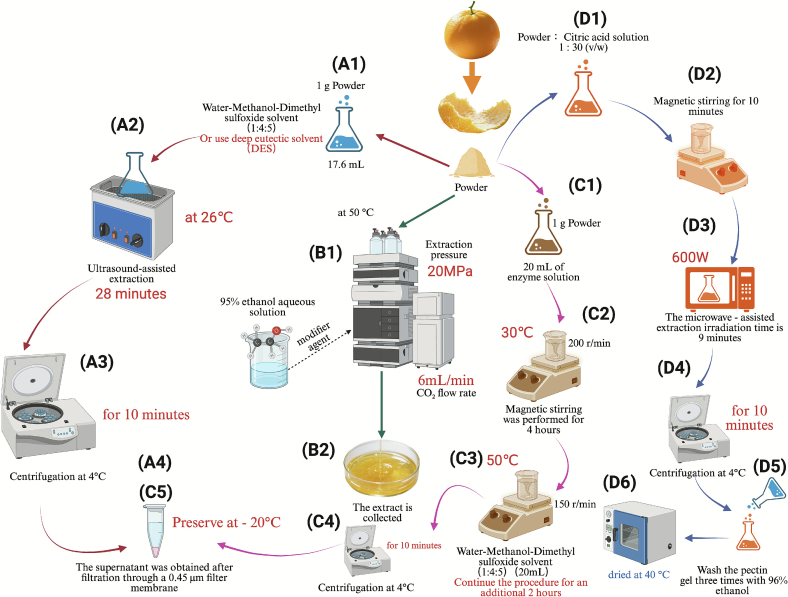
Green extraction technology. Ultrasound-assisted extraction with deep eutectic solvent (Ratanasongtham et al., 2024; Wang et al., 2023) (A); Supercritical fluid extraction (Chen & Huang, 2016) (B); Enzyme-assisted extraction (Ruviaro et al., 2019) (C); Microwave-assisted extraction (Hossain et al., 2024) (D). (For interpretation of the references to colour in this figure legend, the reader is referred to the web version of this article.)

Innovations in extraction technology have steadily advanced in recent years, driven by the need for more sustainable, efficient, and environmentally friendly methods. Traditional extraction techniques, which rely heavily on organic solvents, have raised environmental and health concerns due to the persistence of toxic residues in the final products. In contrast, green extraction technologies, which have emerged as a response to these issues, focus on enhancing extraction efficiency while minimizing environmental impact and energy consumption. These technologies utilize physical field-assisted extraction, biocatalysis, and green solvents, replacing traditional solvents with more sustainable alternatives. The importance of green extraction methods lies not only in their technical advantages, such as increased efficiency and selectivity, but also in their potential to transform industrial production, making it more sustainable and aligned with environmental goals (Guler et al., 2024; Ummat et al., 2021). Various green extraction techniques, as shown in Fig. 3, have been developed and continue to evolve.

### 3.1 Supercritical fluid extraction

Supercritical fluid extraction (SFE), particularly when utilizing supercritical carbon dioxide (SC-CO₂), has emerged as a promising green extraction technology. A substance reaches its supercritical state when both pressure and temperature exceed its critical values, resulting in a supercritical fluid, a single non-condensable phase where the liquid-gas interface disappears, creating a uniform fluid with both liquid and gas-like properties (Amaral et al., 2017; Mohammadi et al., 2024). This unique state allows for the precise tuning of fluid density, enhancing its capacity to extract compounds from complex matrices while minimizing toxic residues. The key advantages of SFE include its high selectivity, safety, efficiency, and rapid mass transfer, making it an ideal method for extracting bioactive compounds without compromising the integrity of sensitive substances (Singh et al., 2021). Moreover, SFE has been effectively applied to extract lipids and bioactive compounds from agricultural waste, such as soybean residues. The incorporation of ethanol as a co-solvent further enhanced the extraction of phenolic compounds and isoflavones, resulting in extracts with notable antioxidant properties (Díaz-de-Cerio et al., 2024).

SFE is versatile and applicable across various industries, particularly for extracting essential oils, lipids, and bioactive compounds from plant materials and agricultural waste. However, process parameters, such as temperature and pressure, must be carefully controlled to optimize efficiency. Moderate temperature increases (40–60 °C) can enhance yields, but excessive heating may degrade sensitive compounds. Pressure control (10–30 MPa) is crucial for enhancing the solubility of CO₂ and improving the extraction of targeted compounds. Additionally, the incorporation of polar solvents, like ethanol or water, as co-solvents can further improve the extraction of polar compounds, increasing yield and selectivity. For instance, the extraction of essential oils from plants like thyme and oregano using SC-CO₂ has demonstrated high efficiency and selectivity (Yousefi et al., 2019). Despite its advantages, SFE does have limitations, such as high equipment costs and the need for specialized modifiers. The lack of a fully mature continuous production process limits its scalability to small-scale or high-value product extractions (Ahangari et al., 2021). Future advancements in optimizing parameters and developing more effective polar co-solvents could expand the applicability of SFE to larger-scale operations.

### 3.2 Enzyme-assisted extraction

Enzyme-assisted extraction (EAE) uses hydrolytic enzymes to degrade cell walls and other cellular components, facilitating the diffusion of solvents into plant or fungal materials to release metabolites. EAE has proven effective in extracting a wide range of bioactive compounds, including proteins, polyphenols, and lipids, from various plant and fungal residues (Lubek-Nguyen et al., 2022). This method offers the benefit of using water or mild solvents, reducing the need for harmful organic solvents and minimizing environmental pollution.

For instance, EAE has been utilized to extract proteins from beet by-products, resulting in a 43% increase in protein yield compared to conventional methods (Akyüz & Ersus, 2021), and it has also been shown to recover higher amounts of bioactive procyanidins from sweet cherry residues, exhibiting enhanced bioactivity (Domínguez-Rodríguez et al., 2021). Additionally, when combined with high hydrostatic pressure, EAE has been used to enhance polyphenol extraction from grape marc, providing higher yields and preserving bioactivity (Teles et al., 2021). EAE also offers additional benefits beyond improving extraction rates. The main advantage of EAE lies in its ability to extract bioactive compounds without exposing them to harsh conditions, such as high temperatures, thereby preserving sensitive components like polyphenols and flavonoids (Bitwell et al., 2023; Dobrincic et al., 2020; Mohan et al., 2022). Future research could focus on developing high-specificity enzymes and optimizing processes to enhance efficiency and reduce costs.

### 3.3 Deep eutectic solvents

Deep eutectic solvents (DES) are a class of green solvents composed of a hydrogen bond acceptor (HBA) and a hydrogen bond donor (HBD), which exhibit a lower melting point than their individual components due to charge delocalization induced by hydrogen bonding (Wang & Lee, 2021). DESs are considered a safer, biodegradable, and cost-effective alternative to traditional solvents, such as ionic liquids, and have found applications in various industrial sectors, including extraction, separation, and materials synthesis (Prabhune & Dey, 2023).

One of the main advantages of DESs is their ability to selectively extract bioactive compounds while being environmentally benign. For example, a natural DES composed of C8 and C10 fatty acids has been used to efficiently extract *β*-carotene from pumpkin, with the extracted *β*-carotene remaining stable for over 180 days (Stupar et al., 2021). Similarly, DESs made from choline chloride and carboxylic acids have been shown to be 15% more efficient than ethanol for extracting phenolic compounds from olive leaves (Pontes et al., 2021). However, DESs do present some challenges, such as high viscosity, which can hinder solute diffusion and prolong extraction times. Additionally, some DESs have high melting points, requiring heating to maintain their liquid state, which can increase energy consumption (Mohan et al., 2022). To overcome these limitations, DESs are often combined with other green extraction technologies, such as UAE, MAE, or enzymatic pre-treatment, which can improve extraction efficiency and reduce processing time (Picot-Allain et al., 2021). In future need to be focused on the development of lower-viscosity DESs, as well as their integration with other green technologies, holds significant promise for improving extraction efficiency and reducing energy costs.

### 3.4 Ultrasound-assisted extraction

Ultrasound-assisted extraction (UAE) offers an eco-friendly and efficient alternative to traditional extraction methods, which often rely on large quantities of organic solvents. UAE utilizes high-frequency sound waves to generate cavitation bubbles in the solvent, which facilitates the release of bioactive compounds from plant or animal matrices. This technology is widely recognized for its high efficiency, low solvent consumption, and ability to extract heat-sensitive compounds (Shen et al., 2023). UAE is particularly effective in extracting a variety of bioactive compounds, including phenolics, polysaccharides, and proteins, from plant-based materials.

A key advantage of UAE is its ability to enhance extraction efficiency while reducing processing time and solvent usage. Furthermore, the method is particularly suitable for extracting heat-sensitive compounds, as it operates at relatively low temperatures. The combination of UAE with DES has further enhanced its extraction efficiency. For example, the combination of choline chloride and glycerol in a 1:2 M ratio has been found to significantly improve phenolic extraction and bioactivity from foxtail millet bran when used with UAE (Zheng et al., 2022). Similarly, UAE has been optimized for the extraction of polysaccharides from Shatian pomelo peel, achieving higher yields and enhanced antioxidant potential compared to traditional hot water extraction methods (Lin et al., 2023). Despite these advantages, UAE does have some limitations, such as the potential for damage to active component molecules due to high ultrasound power, which can lead to degradation. Additionally, localized high temperatures generated during ultrasound may reduce extraction yields and antioxidant capacity by causing component oxidation or structural isomerization (Lin et al., 2021). Future research should optimize ultrasound power and duration, and explore UAE's integration with other green solvents to boost extraction efficiency and preserve bioactive compound integrity.

### 3.5 Microwave-assisted extraction

Microwave-assisted extraction (MAE) enhances extraction efficiency by utilizing microwave radiation to generate heat through molecular interactions within the solvent-sample mixture. This technology accelerates the extraction of bioactive compounds by inducing rapid heating, which results in efficient solvent penetration into plant cell walls, leading to the rapid release of bioactive substances. MAE is particularly effective for extracting phenolic compounds, alkaloids, and essential oils from plant materials (Pandit et al., 2015; Wang et al., 2018).

One of the key advantages of MAE is its ability to achieve volumetric heating, which significantly improves extraction efficiency and reduces processing time. For example, MAE has been successfully used to extract phenolic compounds from pomegranate and banana peels, demonstrating its efficacy in recovering bioactive substances in a sustainable manner (Shijarath et al., 2024). MAE is often combined with DES to further improve efficiency. A study showed that MAE, when coupled with a choline chloride and 1,4-butanediol-based DES, effectively extracted polysaccharides from kelp, yielding high antioxidant activity (Shang et al., 2021). Similarly, another study utilized MAE in conjunction with natural DES to recover bioactive compounds from hazelnut residues, resulting in antioxidant capacity two to three times greater than that achieved with ethanolic extracts, further demonstrating the synergy between MAE and DES in enhancing extraction outcomes (Bener et al., 2022). The rapid temperature increase facilitated by MAE enhances solvent penetration into plant cell walls, promoting the release of target compounds and improving extraction efficiency while reducing solvent usage. However, MAE has certain limitations, particularly in extracting heat-sensitive and non-polar compounds, where excessive heating may cause degradation or low solubility (Chaves et al., 2020; Zhang et al., 2020). Future work should focus on overcoming these limitations by optimizing MAE for heat-sensitive and non-polar compounds, and enhancing its effectiveness through better combinations with green solvents like DES.

Conventional extraction technologies for bioactive compounds face several limitations, including high energy consumption, thermal degradation of heat-sensitive components, risks of organic solvent residues and pollution, low extraction efficiency, and prolonged processing times. In contrast, green extraction technologies such as UAE, MAE, DES, SFE, and EAE have achieved significantly improved extraction efficiency, effective preservation of bioactive components, and a substantial reduction in environmental footprint through non-thermal or mild processes, environmentally friendly solvents, and targeted mechanisms of action. They also offer advantages including low solvent consumption and short operation cycles (More et al., 2022; Nayak et al., 2024). Future research should focus on optimizing operational parameters, exploring synergistic combinations of multiple techniques, and advancing large-scale applications to establish highly efficient, sustainable extraction processes for bioactive compounds in functional foods, aquafeeds, and pharmaceutical applications.

## 4 Application of green extraction technology in citrus waste

Various green extraction technologies exhibit distinct advantages and limitations (Table 1), and no single approach is universally applicable to the full spectrum of bioactive compounds present in citrus by-products. SFE exhibits high selectivity toward low-polarity compounds, making it an optimal choice for extracting terpenoids. This technology significantly enhances extraction efficiency and avoids solvent residues; however, it demonstrates limited effectiveness for polar compounds such as phenolic acids (Domínguez-Rodríguez et al., 2025; Ndayishimiye & Chun, 2017; Su et al., 2022). In contrast, EAE efficiently recovers phenolic compounds under mild conditions, preserving bioactivity, yet the high cost of enzymes and long processing times remain notable drawbacks (Barbosa et al., 2021; Multari et al., 2021; Yeh & Lai, 2022). When DES are employed as an extraction medium, they enable the simultaneous extraction of multiple polypolar constituents, including flavonoids, phenolic acids, and essential oils, with the resulting extracts exhibiting excellent antioxidant and anticholinesterase activities. Nevertheless, high solvent costs and challenges in viscosity control and recovery hinder large-scale implementation (Domínguez-Rodríguez et al., 2025; Xu et al., 2019; Zhang et al., 2024). UAE offers remarkable extraction efficiency and enhanced antioxidant potential but requires precise control of processing parameters (Khandare et al., 2021; Li et al., 2022; Panwar et al., 2023), while MAE reduces time and energy consumption with broad solvent compatibility, yet its efficiency for phenolic acids is relatively limited (Cigeroglu et al., 2021; García-Martín et al., 2023; Rodsamran & Sothornvit, 2019). Overall, the choice of extraction technology should align with the specific bioactive compounds targeted. SFE and UAE are widely used in large-scale production but involve high initial equipment costs, whereas DES and EAE face limitations in consumable recovery. Currently, the synergistic integration of multiple green extraction techniques has emerged as a promising strategy to enhance overall extraction efficiency, performance, and sustainability.

**Table 1:** Comparison of green extraction technologies for bioactive compounds derived from citrus by-products.

Green Extraction Technology	Citrus Varieties	Bioactive Compounds	Scale	Advantages	Limitations	References
SFE	-Pomelo -Grapefruit -lemon -Yuzu	-Flavonoids -Coumarins -Organic acids -Terpenoids -Carotenoid	Industry	-Efficiently extracts low-polarity small molecules and enriches bioactives such as flavonoids and coumarins with proven antioxidant, anti-inflammatory and antimicrobial activities -The SFE extract exhibits significantly stronger anticholinergic activity than those obtained by other methods -CO₂ solvent requires no separate removal step and is recyclable, reducing solvent costs -Reducing extraction time can help lower time costs in large-scale production	-Unable to efficiently extract polar compounds, such as phenolic substances found in citrus by-products -Strict control of pressure and temperature is required, as parameter fluctuations can easily reduce extraction efficiency, thereby increasing operational complexity in large-scale production -High equipment costs during the initial investment phase	(Domínguez-Rodríguez et al., 2025; Ndayishimiye & Chun, 2017; Su et al., 2022)
EAE	-Grapefruit -Lemon -Sweet orange	-Flavonoids -Phenolic acids -tangeretin -Pectin	Laboratory	-Significantly improved the extraction efficiency of phenolic compounds from citrus by-products -The extraction process is carried out under relatively mild conditions, which largely preserves the integrity of the bioactive components -Free of toxic organic solvent residues, meeting food-grade extract standards	-The procurement cost of enzymes is higher than that of traditional solvent extraction -Enzymatic hydrolysis usually requires long reaction times, resulting in low output per unit time	(Barbosa et al., 2021; Multari et al., 2021; Yeh & Lai, 2022)
DES	-tangerine -pomelo	-Flavonoids -Pectin -Essential oil -Phenolic acids	Laboratory	-Enables the simultaneous extraction of bioactives spanning a wide polarity range, overcoming the selectivity limitation of traditional solvents -Using choline chloride as the hydrogen-bond acceptor paired with natural hydrogen-bond donors offers good biodegradability and low toxicity -Enables the simultaneous extraction of compounds possessing both antioxidant and anticholinergic activities, with both activities significantly higher than those achieved by conventional ethanol extraction	-The component ratio of the composite DES is highly sensitive; even slight deviations can significantly reduce extraction efficiency -Due to the high viscosity of DES, large-scale production usually requires coupling with additional extraction techniques to achieve optimal performance -The inherent difficulty in solvent recovery increases the pressure on large-scale production	(Domínguez-Rodríguez et al., 2025; Xu et al., 2019; Zhang et al., 2024)
UAE	- Tribute citrus - Sweet lime	-Essential oil -Monoterpenoids -Pectin	Industry	-High extraction efficiency achieves dual optimization in both time and yield -The bioactives recovered from citrus by-products exhibit higher purity and superior antioxidant performance	-Extraction performance is highly parameter-sensitive; any deviation from the optimum leads to a marked drop in yield -High equipment costs during the initial investment phase	(Khandare et al., 2021; Li et al., 2022; Panwar et al., 2023)
MAE	-Grapefruit -lime -Bitter orange	-Naringin -Phenolic compounds -Phenolic acids -Flavonoids	Industry	-Delivers clear time- and energy-saving advantages in extraction efficiency -Broad solvent compatibility, readily meeting the extraction demands of various flavonoids	-Insufficient recovery of phenolic acids and no evident edge over conventional methods -Highly sensitive to parameters, requiring precise temperature control	(Cigeroglu et al., 2021; García-Martín et al., 2023; Rodsamran & Sothornvit, 2019)

SFE = Supercritical fluid extraction; EAE = Enzyme-assisted extraction; DES=Deep eutectic solvent; UAE = Ultrasound-assisted extraction; MAE = Microwave-assisted extraction.

Despite current minor limitations, green extraction technologies have proven to be highly effective in extracting bioactive compounds from citrus waste (Supplementary Table S1), offering an environmentally friendly alternative to traditional methods. Advanced techniques such as SFE, MAE, and UAE have been increasingly applied to citrus waste, resulting in high yields of essential oils, pectin, bioactive compounds, and dietary fiber, all while minimizing the use of toxic solvents and reducing environmental impact.

For example, MAE has been used to extract pectin from Kinnow (*Citrus reticulata*) peels. Under conditions of 110 °C and pH 2.2, the extracted pectin yielded an equivalent weight of 833 mg, with a methoxyl content of 7.44 %, a degree of esterification of 66.67 %, and a galacturonic acid content of 63.15 %. These values reflect higher purity compared to commercial citrus pectin (Duggal et al., 2024). Another study explored the use of solvent-free microwave-assisted gravity station to extract essential oil from lemon (*Citrus limon*) leaves. At 110 °C and 300 W microwave power for 50 min, the essential oil yield reached 2.5 %, exhibiting stronger antioxidant capacity than butylated hydroxytoluene (Yeasmin et al., 2024). Similarly, SFE has also demonstrated effectiveness in extracting essential oils from citrus peels. A study showed that under optimized conditions of 317.51 min at 74.85 °C and a solvent-to-sample ratio of 4:1, SFE successfully extracted key compounds like limonene, *α*-pinene, and *β*-myrcene, which exhibited strong antioxidant and antimicrobial properties (Ling Felicia et al., 2024).

The combination of green extraction techniques, such as integrating UAE with EAE, is also gaining popularity. For example, UAE was used to extract dietary fiber from Kinnow peels, yielding 52.042 ± 0.862 % dietary fiber. When sequential enzymatic treatment was applied, this yield increased to 60.974 ± 0.827 %, surpassing the yield of single extraction methods. The extracted dietary fiber demonstrated superior water-holding, oil-holding, and glucose adsorption capacities (Kaur, Panesar and Chopra, 2023a, Kaur, Panesar and Chopra, 2023b). Moreover, a combination of supercritical CO₂ extraction (SC-CO₂) and natural DES-assisted UAE was utilized to extract bioactive terpenoids and phenolic compounds from three citrus peels. Direct UAE-DES extraction resulted in the highest phenolic diversity, with naringin found in grapefruit (*Citrus paradisi*) peels and hesperidin in lime (*Citrus aurantiifolia*) and lemon peels as the main compounds. SC-CO₂ pretreatment before UAE-DES extraction did not improve phenolic recovery but increased the anticholinergic capacity (Domínguez-Rodríguez et al., 2025).

In conclusion, while individual green extraction technologies have their limitations, their combination offers a superior solution for processing citrus waste. Compared to traditional methods like solution extraction and Soxhlet extraction, these combined methods are safer, more reliable, and more efficient in terms of extraction capacity and yield.

## 5 Role of citrus waste-derived bioactive compounds in aquaculture

**Fig. 4:**
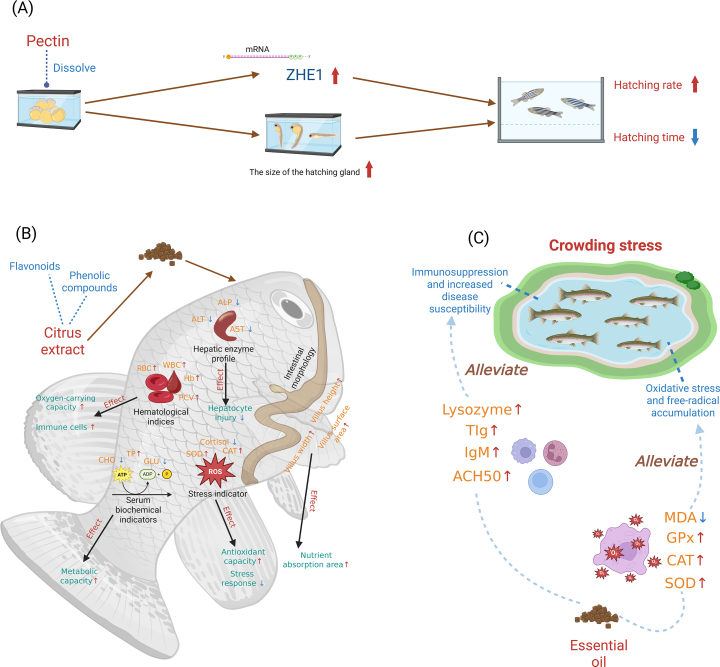
Technological route for applying bioactive nutrient products from citrus to fish. Promotion of zebrafish egg hatching by pectin (Dananjaya et al., 2020) (A); Physiological improvement in fish by phenolic compounds extracted from citrus waste (Silva et al., 2024) (B); Alleviation of crowding stress in fish by citrus essential oils (Chekani et al., 2021) (C). ZHE1 = Zebrafish Hatching Enzyme 1; ALT = Alanine aminotransferase; AST = Aspartate aminotransferase; ALP = Alkaline phosphatase; RBC = Red blood cell count; WBC = White blood cell count; Hb = Hemoglobin; PCV = Packed cell volume; CHO = Total cholesterol; TP = Total protein; GLU = Glucose; TIg = Total immunoglobulin; IgM = Immunoglobulin M; ACH50 = Alternative complement hemolytic activity (50 %); MDA = Malondialdehyde; GPx = Glutathione peroxidase. (For interpretation of the references to colour in this figure legend, the reader is referred to the web version of this article.)

Citrus by-products, such as peels, seeds, and pulp, are rich in bioactive compounds and hold substantial potential for aquaculture applications; however, these materials are frequently discarded, contributing to environmental pollution and representing a significant underutilization of valuable functional resources (Wang et al., 2023). The composition and content of bioactive constituents vary markedly among by-products derived from different citrus varieties, directly influencing their stability for applications. As summarized in Table 2, lemon by-products are particularly rich in pectin, offering high extraction yields and elevated galacturonic acid purity, although their potential for essential oil recovery is limited and the content of the key compound *d*-limonene is comparatively low (Durmus & Kilic-Akyilmaz, 2023; El Fihry et al., 2022; Putri et al., 2022). In contrast, orange by-products exhibit superior potential for essential oil extraction and are characterized by high *d*-limonene content (Fakayode & Abobi, 2018; Farahmandfar et al., 2020; Jridi et al., 2019). Pomelo by-products generally demonstrate lower overall content and purity of bioactive compounds compared to other common citrus species, whereas grapefruit displays a more balanced profile across various bioactive metrics (Ahmed et al., 2024; Armenta et al., 2023; Hung et al., 2020; Van Hung et al., 2021; Zhang et al., 2024). Notably, reported values for total phenolic and flavonoid contents vary significantly, reflecting not only varietal differences but also the strong influence of extraction and processing methodologies. Among all citrus fruit by-products, peels are of particular importance due to their high concentrations of phenolic compounds, essential oils, pectin, and other functional constituents (Kaur, Panesar and Chopra, 2023a, Kaur, Panesar and Chopra, 2023b). These compounds have been shown to confer multiple physiological benefits in aquatic organisms, including enhanced growth, immunity, and stress tolerance (Kesbiç et al., 2022). As illustrated in Fig. 4, citrus-derived bioactive compound such as pectin, phenolic compounds, and essential oils exert multidimensional effects on fish physiology by promoting embryonic development, improving metabolic and antioxidant capacities, alleviating environmental stress, and strengthening immune defenses (Chekani et al., 2021; Dananjaya et al., 2020; Silva et al., 2024). These observations are further supported by the findings summarized in Table 3, which highlight the functional value of citrus waste-derived bioactive compounds for sustainable aquaculture applications.

**Table 2:** The extraction potential of bioactive substances from by-products of different citrus varieties.

Citrus Species	Pectin Yield (%)	Galacturonic Acid (%)	Essential Oil Y yield (mL/kg dry weight)	*d*-Limonene Content (%)	Total Phenolic Content (mg GAE/g dry weight)	Total Flavonoid Content (mg RE/g dry weight)	References
Grapefruit (*Citrus paradisi*)	11.68–17.16	76.19	6.75–18	80.18–95.53	4.09–8.47	4.04–5.15	(Ahmed et al., 2024; Armenta et al., 2023; Putri et al., 2022)
Sweet orange (*Citrus sinensis*)	15.28–16.01	79,0	5.7–32.4	89.12–95.40	8.68–10.07	5.57–6.30	(El Fihry et al., 2022; Fakayode & Abobi, 2018; Jridi et al., 2019; Putri et al., 2022)
Lemon (*Citrus limon*)	16.29–16.61	75.55–82.0	7.74	73.04	3.14–3.76	2.25–4.56	(Durmus & Kilic-Akyilmaz, 2023; El Fihry et al., 2022; Putri et al., 2022)
Bitter orange (*Citrus aurantium*)	8.74–10.51	86.0	10–40	81.19–88.70	6.27–12.80	10.84–16.46	(Ahmed et al., 2024; Farahmandfar et al., 2020)
Pomelo (*Citrus maxima*)	6.5–9.0	66.6	19–20.9	63.06–91.07	4.08–7.50	0.93–2.29	(Hung et al., 2020; Van Hung et al., 2021; Zhang et al., 2024)

**Table 3:** The effect of bioactive compounds derived from citrus on fish.

Bioactive Compounds	Types of Citrus Fruits	Extracted Citrus Parts	Fish Species	Dose	Outcomes	Reference
Pectin	Pomelo (*Citrus maxima*)	Peel	Zebrafish (*Danio rerio*)	0 (control), 50, 100, 200, 300 μg/Ml	Optimal dose at 100 μg/mL: Significantly increased hatching rate; High doses (200–300 μg/mL) caused mortality and reduced hatching rate.	Dananjaya et al. (2020)
Pectin	Orange (*Citrus sinensis*)	Peel	Common carp (*Cyprinus carpio*)	0 (control), 0.5, 1, 2 % in diet	Optimal dose at 1 %: Significantly improved WG, specific growth rate SGR, and FCR were observed, accompanied by the highest SPA and CAT activities; High-dose (1 %–2 %) supplementation significantly increased skin mucus lysozyme activity.	Hosseini et al. (2020)
Pectin	Citrus	pulp	Jundia catfish (*Rhamdia quelen*)	0 (control), 5, 10 g/kg diet	Optimal dose at 5 g/kg: Improved WG and FCR; At 10 g/kg, trypsin and chymotrypsin activity were higher.	Goulart et al. (2017)
Flavonoids, polyphenols.	Lemon (*Citrus limon*)	Peel	*Rohu* *(Labeo rohita*)	0 (control), 2.5, 5.0, 10.0, 20.0 g/kg diet	Optimal dose at 5.0 g/kg: Highest WG (7.87 ± 0.415 g) and lowest FCR (2.10 ± 0.252); 20.0 g/kg: Elevated lipase and antioxidant enzymes (SOD and CAT), but reduced growth efficiency.	Tejaswini et al. (2024)
Phenolic compounds, polysaccharide.	Lemon (*Citrus limon*)	N/A	Nile tilapia (*Oreochromis niloticus*)	0.0 (control), 0.2, 0.4, 0.8, 1.6, 3.2 g/kg diet	Optimal dose at 1.6 g/kg: Improved final weight (85.58 ± 1.36 g), weight gain (72.46 ± 1.35 g), feed intake (99.80 ± 1.33 g), and FCR (1.38 ± 0.02); 0.8–3.2 g/kg: Reduced mortality after *Aeromonas hydrophila* infection; decreased plasma AST activity post-challenge.	Silva et al. (2024)
Phenolic compounds, polysaccharide.	Lemon (*Citrus limon*)	N/A	Striped catfish(*Pangasius hypophthalmus*)	0.0 (control), 0.2, 0.4, 0.8, 1.6, 3.2 g/kg diet	Optimal dose at 0.4 g/kg: Highest weight gain (60.81 ± 2.10 g), lowest FCR (0.55 ± 0.02), increased erythrocytes (2.89 ± 0.36 × 106 μL^−1^) and MCV (145.19 ± 19.67 fL); 3.2 g/kg: Elevated total leukocytes (60.07 ± 5.79103 μL), lymphocytes (22.68 ± 3.83103 μL) and monocytes (3.79 ± 0.52103 μL), but reduced growth.	Macedo et al. (2023)
Phenolics, flavonoids.	Grapefruit (*Citrus paradisi*)	Peel	Caspian white fish (*Rutilus frisii kutum*)	0 (control), 6.25, 12.5, 25 mg/kg diet	Optimal dose at 25 mg/kg: Highest WG, SGR, and lowest FCR, along with improved hematological parameters and intestinal villus surface area.	Samavat et al. (2019)
Essential oil	Orange (*Citrus sinensis*)	Peel	Tambaqui (*Colossoma macropomum*)	200, 400, 800 mg/L	The optimal dosage of 400 mg/L orange essential oil achieved the best weight gain (514.59 ± 1.85 mg) and FCR (1.86 ± 0.02), while significantly increasing thrombocyte count (11.03 ± 0.30 Cell μL^−1^ × 103) and RBC (1.56 ± 0.02 Cell μL^−1^ × 106) in fish; Dietary supplementation with orange essential oil also notably enhanced resistance against *Aeromonas hydrophila* infection.	Pereira Jr. et al. (2025)
Essential oil	Bitter orange (*Citrus aurantium*)	Peel	Common carp (*Cyprinus carpio*)	0.0 (control), 0.25, 0.50, 1.0, 1.5 %	Optimal dose at 0.25 %: Enhanced SGR (2.71 ± 0.07 % day^−1^) and decreased FCR (0.71 ± 0.03); Histological alterations in the liver and intestine at high doses; Increased expression of growth-related genes (*GH* and *IGF-1*) and immune-related genes (*TNF-α, IL-1β*, *IL-8*) at low doses.	Acar et al. (2021)
Essential oil	Bergamot (*Citrus bergamia*)	Peel	Nile tilapia (*Oreochromis niloticus*)	0.0 (control), 0.5, 1.0, 2.0 %	The optimal dose of 0.5 %: significantly increased SGR (5.75 ± 0.11 % day^−1^) and decreased FCR (0.91 ± 0.02); HGB (9.18 ± 0.56 g/dL), HCT (36.56 ± 4.94 %), and RBC (2.84 ± 0.35106cell/mm^3^) were significantly increased; Glucose (61.34 ± 5.54 mg dL^−1^), cholesterol (74.88 ± 9.83 mg dL^−1^), and triglycerides (73.59 ± 8.39 mg dL^−1^) were significantly reduced, total protein (4.72 ± 0.76 g dL^−1^) increased; GPT (19.32 ± 2.17 U L^−1^) and LDH (203.99 ± 15.67 U L^−1^) activities were significantly decreased.	Kesbic et al. (2020)
Essential oil	Lemon (*Citrus limon*)	Peel	Rainbow trout (*Oncorhynchus mykiss*)	0, 0.82 μg/L DMN (deltamethrin),200, 400, 600 mg/kg diet +0.82 μg/L DMN	The optimal dose of 400 mg/kg: owered FCR (1.42 ± 0.07) and heightened SGR (0.79 ± 0.06 % day^−1^); RBC (1.24 ± 0.02106 mm3), Hb (7.64 ± 0.04 g/dL), and Hct (46.00 ± 1.00 %) levels were restored to near-control levels.	Resketi et al. (2021)
Essential oil	Bergamot (*Citrus bergamia*)	Peel	European sea bass (*Dicentrarchus labrax*)	0 %, 0.5 %, 1 %, 2 % in diet	Optimal dose at 0.5 %–1 %: improves growth and feed efficiency in European sea bass juveniles; enhances antioxidant enzymes (SOD and GPx) and immune parameters (lysozyme); Higher doses (2 %) negatively impact growth.	Acar et al. (2019)

WG = Weight Gain; SPA = Serum Protease Activity; CAT = Catalase; FCR = Feed Conversion Ratio; AST = Aspartate Aminotransferase；MCV = Mean Corpuscular Volume; SOD = Superoxide Dismutase; RBC = Red Blood Cell Count; SGR = Specific Growth Rate; HCT = Hematocrit; GPT = Glutamic-Pyruvic Transaminase; LDH = Lactate Dehydrogenase; ALP = Alkaline Phosphatase.

### 5.1 Pectin

Pectin, a polysaccharide abundant in the cell walls and intercellular spaces of higher plants, primarily consists of a linear chain of *D*-galacturonic acid units connected by *α*-1,4-glycosidic bonds (Liu et al., 2021). Traditionally, pectin is used as a food additive due to its thickening and gelling properties, but its applications have extended into medical fields such as drug delivery, wound healing, and tissue engineering (Dananjaya et al., 2020; Singhal & Hulle, 2022). Citrus pectin, which contains a high proportion of methyl esters, stands out due to its superior emulsifying capabilities compared to other forms of pectin (Zhao et al., 2020). Due to its bioactive properties, citrus pectin has gained increasing attention for its potential in aquaculture, where it contributes to fish health, growth, and immune function.

Several studies have examined the effects of citrus pectin on fish, demonstrating its positive impact on growth and immune function. For instance, pomelo peel-derived pectin was tested on zebrafish embryos (*Danio rerio*), where varying concentrations of pectin (0, 50, 100, 200, 300 μg/mL) were evaluated for their effects on embryonic mortality, hatching rate, and *ZHE1* gene expression. At 100 μg/mL, pectin significantly increased the hatching success to 96.6 %, compared to 66.6 % in the control group (*P* < 0.05). However, higher concentrations adversely affected embryo viability, indicating a narrow effective dose range (Dananjaya et al., 2020). Extending this research to common carp, orange peel pectin was incorporated into isonitrogenous diets at 0 %, 0.5 %, 1 %, and 2 % for 8 weeks. The 1% pectin group exhibited the most significant improvements in growth, including a 29 % increase in weight gain (WG), a 21% improvement in feed conversion ratio (FCR), and a 30–40 % rise in skin mucus lysozyme activity, alongside a 25 % increase in serum catalase levels. These results highlight the beneficial effects of orange peel pectin on carp, particularly in promoting mucosal immunity and oxidative balance (Hosseini et al., 2020). Furthermore, *Rhamdia quelen* (Jundia catfish) also showed improved growth when fed diets containing citrus pulp-derived pectin. Pectin supplementation at 5 g/kg and 10 g/kg led to weight gains of 47.3 % and 46.4 %, respectively, compared to 42.9 % in the control group. Notably, the 5 g/kg group showed lower enzyme activities, while higher pectin levels increased digestive enzyme secretion, suggesting a compensatory mechanism to maintain digestion efficiency (Goulart et al., 2017).

The regulatory effects of citrus pectin on fish growth and immune response exhibit pronounced species- and life-stage specificity. Zebrafish embryos, owing to their immature digestive and immune systems, display heightened sensitivity to pectin supplementation, with elevated doses posing potential survival risks. In contrast, adult omnivorous freshwater species such as common carp and jundia catfish possess well-developed intestinal enzyme systems adapted to the digestion of plant-derived polysaccharides, enabling them to maintain metabolic homeostasis through modulation of digestive enzyme activity and mucosal immune responses (Kumar et al., 2005). These contrasting responses reflect the combined influence of developmental stage and intestinal physiological specialization, underscoring the importance of species- and age-appropriate pectin dosage in aquaculture applications.

### 5.2 Phenolic compounds

Phenolic compounds, widely distributed in plants as secondary metabolites, are well-known for their antioxidant properties. These bioactive compounds are commonly classified into simple phenols and polyphenols, with flavonoids (such as flavonols, anthocyanins, isoflavones, and flavones) being the most abundant class found in plant tissues (Khoddami et al., 2013; Xu et al., 2020). Citrus fruits, particularly the peels, are rich in phenolic compounds, which have drawn increasing attention in aquaculture for their potential to improve fish health, growth, and immunity. These compounds not only possess antioxidant properties but also show promise in anti-inflammatory, antimicrobial, and immune-boosting roles, making them valuable for enhancing the sustainability and productivity of aquaculture.

Phenolics have significant roles in aquaculture. Lemon, a rich source of bioactive compounds such as flavonoids and polysaccharides, identified through nuclear magnetic resonance (NMR) and mass spectrometry (MS), has shown potential as a functional feed additive in aquaculture. In a 60-day trial, Nile tilapia was fed diets containing varying concentrations of lemon extract (0.0, 0.2, 0.4, 0.8, 1.6, and 3.2 g/kg) and subsequently challenged with *Aeromonas hydrophila*. The 1.6 g/kg group demonstrated significantly improved specific growth rate to 3.12 ± 0.03 % day^−1^, elevated hemoglobin concentration (17.82 ± 0.45 g dL^−1^), and over 80 % post-infection survival, indicating enhanced immune response and disease resistance. However, the highest dose (3.2 g/kg) negatively impacted growth, highlighting the importance of dose optimization when using lemon extract in aquafeeds (Silva et al., 2024). Lemon extract has demonstrated beneficial effects beyond Nile tilapia, showing promise in *Pangasius hypophthalmus* (striped catfish) as well. Using the same dietary concentrations as in the previous study (0.0, 0.2, 0.4, 0.8, 1.6, and 3.2 g/kg), lemon extract was tested on striped catfish over a 90-day period. The 0.4 g/kg dose proved optimal for promoting specific growth rate (3.99 ± 0.07 % day^−1^), with significant improvements in hemoglobin concentration (9.78 ± 0.32. dL^−1^) and mean corpuscular volume (145.19 ± 19.67 fL). While the highest dose (3.2 g/kg) led to an increase in leukocyte levels, it had a suppressive effect on growth performance. Despite this, lemon extract at various levels enhanced resistance to *Aeromonas hydrophila*, reinforcing its potential as an immune-boosting feed additive across different fish species (Macedo et al., 2023). Thus, low-dose lemon extract in aquaculture feeds can promote fish growth and disease resistance. The high phenolic content in lemon peels makes them highly valuable for the aquaculture industry. Further research on lemon peel extract has shown its potential to enhance the growth and metabolic regulation of *Labeo rohita* (rohu) in cold water aquaculture (18 ± 1 °C). Bioactive compounds were extracted using 80 % ethanol, with antioxidant potential evaluated via DPPH radical scavenging, total phenolic content (TPC), and FRAP assays, peaking at 25 mg/L. Experimental diets containing 0.0, 2.5, 5.0, 10.0, and 20.0 g/kg of the extract were fed to fish for 8 weeks. The 5.0 g/kg dose showed the greatest improvements in weight gain (7.87 ± 0.415 g), protease (0.21 ± 0.311 millimole tyrosine/mg protein), and lipase (0.36 ± 0.271/mg protein) activities. The 10.0 g/kg group exhibited elevated AST (7.01 ± 0.24 nmol oxaloacetate/mg protein) and ALT (3.08 ± 0.12 nmol sodium pyruvate/mg protein), suggesting increased liver activity. However, the 20.0 g/kg dose enhanced antioxidant enzymes but also induced oxidative stress, impairing growth. (Tejaswini et al., 2024). Research has also extended to the peels of other citrus fruits, such as grapefruit, due to their rich content of phenolic and flavonoid compounds, known for their antioxidant and antibacterial properties. A study on juvenile Caspian white fish (*Rutilus frisii kutum*) evaluated the effects of grapefruit peel ethanol extract at doses of 0, 6.25, 12.5, or 25 mg/kg in isonitrogenous diets over a 60-day period. The highest dose (25 mg/kg) led to the most favorable outcomes, including a superior specific growth rate (SGR) of 3.05 ± 0.7 % day^−1^, increased white blood cells (17.72 ± 0.46 × 10^3^/μL), and hemoglobin (8.71 ± 1.12 dL^−1^) levels. Additionally, AST (63.42 ± 2.96 U L^−1^) and ALT (8.97 ± 1.54 U L^−1^) enzyme levels were reduced, while antioxidant activity was significantly elevated. These results demonstrate the potential of citrus peel-derived phenolics as effective feed additives for enhancing fish health when used at optimal levels (Samavat et al., 2019).

The biological effects of citrus phenolic compounds are remarkably species-specific, with their regulatory roles in growth rate, immune modulation, and detoxification capacity showing distinct differentiation based on fish physiological traits and environmental adaptability. Warm-water omnivorous species such as Nile tilapia and striped catfish display high metabolic enzyme activity and efficient phenolic bioavailability; at appropriate supplementation levels, these species show synchronized growth enhancement alongside increased resistance to *Aeromonas hydrophila*, reflected by improved immune-related hematological indices. Nevertheless, tolerance thresholds for phenolics differ markedly among species. In contrast, cold-adapted freshwater fish (e.g., rohu) have relatively low metabolic rates; although high phenolic dose can stimulate detoxification-related enzyme activities, they may concurrently suppress growth, highlighting the influence of thermal adaptation on phenolic functional balance. Caspian white fish, another omnivorous freshwater species, exhibit synergistic improvements in growth, immune response, and detoxification capacity when administered higher phenolic extract doses, further underscoring the importance of species-specific adaptation. Collectively, citrus polyphenol-rich extracts hold considerable promise as functional aquafeed additives. However, current studies rely predominantly on conventional ethanol extraction, with limited systematic comparison or optimization of alternative extraction strategies. The adoption of green extraction technologies therefore represents a critical and timely direction for future research. Moreover, mechanistic investigations into the molecular pathways governing phenolic-mediated immune and detoxification responses remain scarce, and differences in effector pathways among fish with varying feeding habits and thermal adaptations have yet to be systematically explored. Addressing these gaps is essential for the precise, standardized, and sustainable application of citrus phenolics in aquaculture nutrition.

### 5.3 Essential oil

Citrus essential oils, primarily extracted from fruit peels through cold pressing, are aromatic, volatile compounds that have found extensive use in the food and pharmaceutical industries (Teigiserova et al., 2021). The primary component of these oils, limonene, constitutes around 90 % of the composition and plays a crucial role in their bioactivity (Maurya et al., 2024). These essential oils are known for their strong antioxidant, antimicrobial, and antifungal properties, and they act as *α*-glucosidase inhibitors, helping regulate blood glucose levels post-meal by slowing carbohydrate breakdown (Tshiyoyo et al., 2025). With advancements in nanotechnology, citrus essential oils have also been formulated into nanoemulsions, which improve their shelf life and preserve bioactivity (Medeleanu et al., 2023). Given their wide-ranging bioactive properties, these oils are being increasingly explored in aquaculture to improve fish health, performance, and disease resistance.

A notable study on orange essential oil (95.2% limonene and 1.8% *β*-pinene as its principal constituents) examined its effects as a dietary additive in Tambaqui (*Colossoma macropomum*), where varying inclusion levels (0, 200, 400, and 800 mg/kg) were tested. The 400 mg/kg dose resulted in the most favorable outcomes, including significant increases in weight gain (514.59 ± 1.85 mg) and immune function, as evidenced by elevated platelet counts (11.03 ± 0.30 Cell μL^−1^ × 10^3^) and lymphocytes (34.06 ± 0.42 Cell μL^−1^ × 10^3^). Additionally, fish in this group showed improved resistance to *Aeromonas hydrophila* infection, suggesting an enhanced innate immune response (Pereira Jr. et al., 2025). Following the success of orange essential oil in aquafeed, bitter orange essential oil has also shown promise in enhancing fish health and performance. In a 60-day trial, juvenile Common carp were fed diets containing 0.0, 0.25, 0.50, 1.0, or 1.5 % of the oil. The 0.25 % inclusion significantly improved specific growth rate (2.71 ± 0.07 % day^−1^) and upregulated genes related to growth (*GH* and *IGF-1*) and immunity (*TNF-α*, *IL-1β* and *IL-8*), without causing any pathological damage. However, higher doses (1.0 % and 1.5 %) led to severe hepatic lipid accumulation and hepatocyte edema, as confirmed by histopathological analysis. These results support the use of bitter orange essential oil as a functional additive at low, non-toxic levels in aquaculture (Acar et al., 2021).

Extending the exploration of citrus-derived essential oils in aquaculture, other varieties such as bergamot (*Citrus bergamia*) and lemon have also demonstrated significant biological benefits across different fish species. Bergamot peel essential oil, widely recognized for its aromatic and bioactive properties, has demonstrated functional potential in aquafeeds. In Nile tilapia, dietary supplementation with 0.0 %, 0.5 %, 1.0 %, or 2.0 % bergamot peel oil over eight weeks revealed that 0.5% was the optimal inclusion level. This dose significantly enhanced SGR (5.75 ± 0.11 % day^−1^), lowered glucose (61.34 ± 5.54 mg dL^−1^) and triglyceride (73.59 ± 8.39 mg dL^−1^) levels, and reduced GPT (glutamic-pyruvic transaminase) and LDH (lactate dehydrogenase) activities to 19.32 ± 2.17 U L^−1^ and 203.99 ± 15.67 U L^−1^ indicative of improved liver health. Additionally, hemoglobin levels and red blood cell counts were significantly increased at both 0.5 % and 1.0 %, suggesting better oxygen transport. However, the highest inclusion (2.0%) impaired digestion and induced mild stress responses (Kesbic et al., 2020). Similar benefits were observed in European sea bass, where dietary inclusion of bergamot peel oil at 0.5 % and 1.0 % for 60 days significantly improved SGR (2.20 ± 0.02 and 2.18 ± 0.03 % day^−1^), reduced blood glucose (111.71 ± 7.32 and 100.76 ± 11.11 mg/dL) and cholesterol (241.51 ± 26.96 and 231.34 ± 15.71 mg/dL) levels, and increased total serum protein (7.56 ± 0.33 and 7.51 ± 0.42 g/dL). Again, the 2.0 % dose impaired growth, reinforcing the need for precise dosage (Acar et al., 2019). Finally, lemon essential oil has been explored for its antioxidant and anti-stress properties in aquaculture. In a study with Rainbow trout exposed to deltamethrin (DMN) stress, dietary supplementation with 400 mg/kg of lemon essential oil improved SGR (0.79 ± 0.06 % day^−1^), normalized red blood cell count (1.24 ± 0.02 × 10^6^/mm^3^), and hemoglobin (7.64 ± 0.04 g/dL), while significantly lowering ALT levels. These results suggest that lemon essential oil can mitigate chemical stress in aquaculture and enhance overall fish health (Resketi et al., 2021).

The physiological effects of citrus-derived essential oils in fish are strongly dose-dependent and species-specific. Among omnivorous freshwater species, such as Nile tilapia and common carp, low to moderate supplementation levels consistently promote growth performance and immune function, whereas excessive doses may lead to adverse effects, including digestive impairment or hepatic lipid accumulation, with tolerance thresholds varying among species. In cold-water freshwater fish, such as rainbow trout, tolerance to essential oils is generally lower, and their primary benefits are associated with enhanced anti-stress responses and detoxification capacity rather than growth promotion. Notably, the euryhaline migratory species European sea bass also demonstrates improved growth and immune responses at low to moderate inclusion levels, while high doses result in growth inhibition. These findings indicate that, despite the currently limited number of studies in marine and brackish aquaculture systems, citrus by-product–derived essential oils possess considerable potential as functional feed additives when applied with species- and dose-specific precision.

This review indicates that the regulatory effects of citrus-derived pectin, phenolics, and essential oils on fish are governed by a multidimensional interaction among compound type, fish physiology, and supplementation dosage. Omnivorous freshwater species show the greatest adaptability to pectin and phenolics, with moderate inclusion levels simultaneously enhancing growth performance and immune function. In contrast, cold-water freshwater fish exhibit lower tolerance thresholds, with physiological responses primarily associated with antioxidant defense and stress mitigation, while evidence for marine species remains limited. Several critical knowledge gaps persist. First, the influence of extract physicochemical properties and solvent polarity on bioavailability in fish has not been systematically elucidated. Second, interactions between citrus-derived bioactive compound and the fish intestinal microbiota remain largely unexplored. In addition, species-specific differences in metabolic and detoxification pathways are poorly understood. Accordingly, future research should adopt an integrated framework encompassing extract characteristics, bioavailability, gut microbiota interactions, and host metabolic responses. Exploring synergistic effects among multiple citrus-derived compounds and expanding investigations to marine species will be essential. Addressing these gaps will provide a robust theoretical foundation for precise dosage formulation and standardized application of citrus by-products in sustainable aquaculture systems across diverse fish species.

## 6 Challenges and future prospectives

**Fig. 5:**
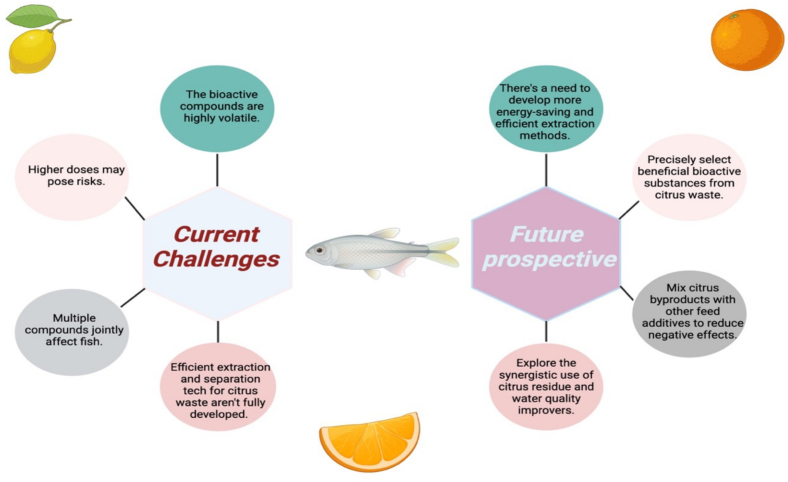
Current challenges and future prospective in the application of citrus waste in aquaculture.

Food waste management is a pressing societal issue, and recycling remains the most sustainable solution. Among food waste, citrus by-products, comprising peels, pulp, and seeds, are generated in large volumes due to high global citrus consumption. These residues are rich in valuable bioactive compounds such as flavonoids, phenolic acids, pectin, vitamin C, and limonene, which hold promising applications across the food, pharmaceutical, industrial, and aquaculture sectors. In aquaculture, the inclusion of citrus waste as a feed additive has shown potential to enhance immune function, antioxidant capacity, and metabolic activity in fish, without compromising growth performance. This dual benefit not only supports sustainable aquaculture but also helps prevent environmental pollution from improper citrus waste disposal. However, as illustrated in Fig. 5, several critical challenges hinder the optimized and large-scale application of citrus by-products. Most current studies rely on direct incorporation of unprocessed citrus waste into aquafeed, often leading to inconsistent and species-dependent outcomes. To translates promising experimental results into reliable industrial practices, future research should prioritize the development of standardized extraction processes and the precise identification and quantification of active bioactive compounds. Moreover, investigations into synergistic combinations of citrus-derived constituents should extend beyond efficacy enhancement to include comprehensive safety evaluations, optimal dosage determination, and long-term stability assessments. Aligning these efforts with regulatory frameworks for novel aquafeed ingredients will be essential for enabling the safe, effective, and scalable utilization of citrus waste-derived bioactives in sustainable aquaculture systems. Furthermore, evaluating the role of citrus waste in improving water quality, such as lowering ammonia‑nitrogen levels, could offer additional ecological benefits. Collectively, these strategies present an opportunity to advance citrus waste valorization through sustainable and innovative aquaculture practices.

## 7 Conclusion

This review highlights the growing interest in utilizing citrus waste, particularly citrus peels and essential oils, as valuable bioactive compounds in aquaculture. Citrus by-products, rich in flavonoids, phenolic acids, essential oils (like limonene), and pectin, offer multiple health benefits for fish, including enhanced growth, immune function, and disease resistance. Various extraction methods, such as ethanol extraction and cold pressing, have been used to obtain bioactive compounds from citrus waste, each method affecting the composition and bioactivity of the resulting extracts. The review also discusses the significance of citrus peel, which has higher concentrations of phenolic compounds, and citrus essential oils, which are rich in limonene, known for its antioxidant, anti-inflammatory, and antimicrobial properties.

Looking ahead, future research should emphasize techno-economic feasibility, logistics of citrus waste streams, and sustainability assessment as interlinked pillars for the practical implementation of citrus by-products in aquaculture. First, green extraction technologies should be further optimized to achieve an effective balance between extraction yield, energy efficiency, and process scalability, thereby ensuring industrial applicability through economically viable protocols. Second, integrated waste stream management strategies should be developed to link citrus processing residues with aquafeed production, while exploring synergistic combinations between citrus-derived bioactive compound and other functional feed additives to establish stable and resilient supply chains. Third, the adoption of quantitative sustainability indicators, such as water quality improvement metrics and life cycle assessment, will be essential for systematically evaluating environmental performance. Finally, expanding validation studies to marine species and commercial-scale aquaculture systems will be critical for confirming the real-world feasibility and impact of citrus waste valorization.

## CRediT authorship contribution statement

**Yu Wang:** Writing – original draft, Software, Formal analysis, Data curation. **Meng-Ze Nie:** Software, Formal analysis, Data curation. **Ping Shi:** Validation, Software, Methodology, Investigation. **Yuan-Sen Liu:** Software, Methodology, Data curation. **Wei-Bing Lan:** Software, Funding acquisition, Data curation. **Zi-Ru Dai:** Resources, Investigation, Conceptualization. **Muhammad Adeel:** Writing – review & editing, Investigation, Data curation. **Hafiz Umer Javed:** Writing – review & editing, Visualization, Supervision, Resources, Conceptualization.

## Funding

This work was financially supported by the Beibu Gulf University Research System (Grant No. 81/23KYQD43, Research Grant: HAFIZ UMER); Guangxi Natural Science Foundation (2023GXNSFBA026244); Scientific Research Basic Ability Improvement Project for Young and Middle-aged Teachers in Guangxi Universities (2023KY0447); Scientific Research Start-up Funding Project for High-level Talents (23KYQD14); Project of Guangxi Science and Technology Courtyards Boosting Rural Revitalization (Construction of Guangxi Pubei Tangerine Peel Science and Technology Courtyard).

## Declaration of competing interest

The authors declare that they have no known competing financial interests or personal relationships that could have appeared to influence the work reported in this paper.

## Data Availability

Data will be made available on request.

## Appendix A Supplementary data

Supplementary material Image 1
