# Supplementary material for: From waste to wellness: Citrus by-products as nutritional and immunological enhancers in aquaculture
Source: Food Chem X. 2026 Jan 12;33:103523. doi: 10.1016/j.fochx.2026.103523 (PMC12853054; doi:10.1016/j.fochx.2026.103523)
Supplement: Supplementary file 1 — Supplementary material [file mmc1.docx]

Supplementary Table S1: The effect of green extraction technologies on citrus waste

| Citrus Varieties | Extracted Compounds | Yield | Extraction Method | Operating Conditions | References |
| --- | --- | --- | --- | --- | --- |
| kinnow (*Citrus reticulata*) peels | pectin | 9.81% | MAE | Temperature: 110°C; pH: 2.2; Time: 10 min; Supernatant to ethanol ratio: 1:2 (v/v) | Duggal et al. (2024) |
| Lemon (*Citrus limon*) leaves | Essential oil | 2.5% | MAE | Time: 50 min; Temperature: 110°C;  Microwave power: 300 W | Yeasmin et al. (2024) |
| Orange (*Citrus sinensis*) peels | Essential oil | 5.08 ± 0.19% | SFE | Time: 317.51 min; Temperature: 74.85 °C, Solvent-to-sample ratio: 4：1 | Ling Felicia et al. (2024) |
| Kinnow (*Citrus reticulata*) peels | Dietary fiber | UAE: 52.042±0.862%; UAE+EAE: 60.974±0.827% | UAE; EAE | Amplitude: 38%; Liquid-to-solid ratio (LSR): 40 mL/g; Temperature: 44°C; Sonication time: 13 min; Enzymatic treatment: Sequential use of α-amylase, protease, amyloglucosidase | Kaur et al. (2023) |
| 1. Grapefruit (*Citrus paradisi*) peel;  2. lime (*Citrus × aurantiifolia*) peel;  3. lemon (*Citrus limon*) peel | Bioactive terpenoids and phenolic compounds | **1. Total phenolic content (TPC):** Grapefruit peel: 39.2 ± 0.7 mg GAE/g, Lime peel: 62 ± 4 mg GAE/g, Lemon peel: 38.3 ± 0.6 mg GAE/g;  **2. Antioxidant capacity (TEAC):** Grapefruit peel: 0.171 ± 0.002 mmol Trolox/g, Lime peel: 0.91 ± 0.06 mmol Trolox/g, Lemon peel: 0.792 ± 0.006 mmol Trolox/g | UAE; SFE; DES | **1. SC-CO**₂**:** Pressure: 200 bar; Temperature: 60°C; Time: 120 min; Solvent: CO₂;  **2. UAE-DES extraction:** DES composition: Grapefruit and lemon peels: Choline Chloride: Tartaric acid (1:2) with 50% water; Lime peel: Choline Chloride: Glycerol (1:2) with 50% water; Ultrasound conditions: 50 min, room temperature | Domínguez-Rodríguez et al. (2025) |

MAE=Microwave-assisted extraction; SFE=Supercritical fluid extraction; EAE=Enzyme-assisted extraction; DES=Deep eutectic solvent; SC-CO₂= supercritical CO₂ extraction.
